# Evidence-Driven Policies for Sustainably Scaling Up Surgical Task-Sharing in Malawi: Comment on "Improving Access to Surgery Through Surgical Team Mentoring – Policy Lessons From Group Model Building With Local Stakeholders in Malawi"

**DOI:** 10.34172/ijhpm.2022.6979

**Published:** 2022-04-13

**Authors:** Desmond T. Jumbam, Ulrick Sidney Kanmounye, Isabelle Citron, Patrick Kamalo

**Affiliations:** ^1^Department of Policy and Advocacy, Operation Smile, Virginia Beach, VA, USA.; ^2^Operation Smile Ghana, Accra, Ghana.; ^3^Operation Smile DR Congo, Kinshasa, Democratic Republic of Congo.; ^4^Royal London Hospital, London, UK.; ^5^Department of Neurosurgery, Queen Elizabeth Central Hospital, Blantyre, Malawi.

**Keywords:** Global Surgery, Task-Sharing, Health Financing

## Abstract

This commentary discusses an article by Broekhuizen et al which assesses policy options for scaling up the SURG-Africa surgical team mentoring program in Malawi to increase access to surgical care. In modeling these scenarios, the authors assess the cost of scaling up surgical teams mentoring and the impacts of scaling the program on district hospitals (DHs) and central hospitals (CHs). The additional costs borne by DHs when increasing surgical volume remains a significant issue identified by the authors and could ultimately determine the success of the program. The piece indirectly advocates for an increased role for task-shifting. The Ministry of Health of Malawi will have to ensure the appropriate governance and regulatory processes are in place to maintain quality and accountability.

We read with interest the article by Broekhuizen et al titled “Improving Access to Surgery Through Surgical Team Mentoring – Policy Lessons From Group Model Building With Local Stakeholders in Malawi.”^1^ Their study assesses scenarios for the continuation and scale-up of the SURG-Africa surgical team mentoring interventions in Malawi. To accomplish this, first, the authors organized two group model-building workshops with actors from district hospitals (DHs) and central hospitals (CHs) to assess factors that could influence the sustainability and replicability of the SURG-Africa surgical mentorship program in Malawi. Second, they model the impact of six different implementation scenarios of the mentorship program. For each scenario, the authors model the changes in referrals from DHs to CHs, financial implications for DHs and funders, and implications in terms of surgical specialist days lost and bed occupancy days averted at CHs.

Our observation is that despite an exponential rise in publications relating to “global surgery,” there remains little evidence-based implementation science to apply and scale evidence-based interventions to improve quality or access to surgical care in low- and middle-income countries (LMICs) where the surgical burden is greatest.^2,3^

In their paper, Broekhuizen et al have tackled this difficult knowledge gap head-on by taking the time and resources to implement and rigorously evaluate an intervention aimed at improving surgical care in a resource-constrained setting.^1^ They have then honestly evaluated its applicability as a nationwide policy intervention, the cost and opportunity cost of implementation to policy-makers and implementers, and highlighted the many potential threats and limitations to its effectiveness. Since the Lancet Commission on Global Surgery publication in 2015, the number of articles relating to “global surgery,” defined as work to improve quality and access to surgical care worldwide, has grown significantly.^2^ However, the majority of these works could be classified as “advocacy” pieces as they spread the research of the Lancet Commission on Global Surgery to a wider audience or highlight new takes on publicly available information.^4,5^ Other studies have focused on descriptive assessments of surgical capacity in LMICs or review articles.^6-8^ Few contain empirical studies assessing the implementation and scale-up of surgical interventions in resource-constrained settings. This is often because acquiring primary data is challenging and expensive, requiring dedication and “hard work,” particularly in resource-constrained environments.^9^

Within the global surgery literature, there has been an emphasis on creating National Surgical, Obstetric and Anaesthesia Plans (NSOAPs) to holistically and systematically strengthen surgical systems.^4^ NSOAPs have also been advocated to avoid the pitfalls of vertical disease programming and to create an overarching strategy to tackle each of the World Health Organization (WHO) health system building blocks (service delivery, personnel, infrastructure and consumables, financing, information technology, leadership, and governance) as it relates to surgical care.^10-12^ Such plans have been created in several countries, including Pakistan, Zambia, Tanzania, Rwanda, and more.^11-14^ However, despite the increased interest from Ministries of Health, particularly in Africa, to develop NSOAPs, there remains a severe lack of evidence-based implementation science on implementing and scaling effective interventions to be included as policy within NSOAPs.^3^ NSOAPs have effectively built coalitions in the surgical community and serve as an advocacy tool to promote the inclusion of surgery in National Health Plans.^11^ However, their effectiveness as truly implementable, effective policy documents is limited by this lack of evidence of what to contain within them. Although their article could be interpreted as dense or difficult to read, it is rigorous analyses such as these by Broekhuizen et al, that takes time and expertise to develop and interpret, that will significantly contribute to the implementation science literature and will form the substrate to take NSOAPs from a tool of advocacy to a credible tool for policy and ultimately tangible improvement in the surgical system.

 In their group modelling exercise, the authors demonstrate the complexities of the healthcare system. The context and threats to the effectiveness of their intervention are then expanded on again in the discursive part of the paper. The number of threats to effective implementation are perhaps evidence of the inherently limited impact of programs that touch only one area of the complex healthcare system, in this case, the area of mentorship of existing personnel. In addition to the threats mentioned in this article, several health system building blocks need to be addressed in a coordinated way if the program is to sustain its impact.

## Financing

The financing piece of the health system is often the most crucial and consequential. The authors’ quote, “*implementers [...] should focus on finding ways to incentivize actors so that new (beneficial) behavior emerge*,” Broekhuizen et al point to a threat to the effectiveness of their intervention being a lack of incentives for both the CHs and the DHs involved.^1^ For 90% of DHs in the southern region (where data is actual and not estimated) and over 50% of all DHs overall, the cost of performing surgeries outweighs the cost of referral and the reimbursement from the government (See Figure 5 of their article).^1^ As such, every additional surgery performed at the hospital is loss-making. This is in the context of many DHs already on the brink of insolvency, relying on donor funds and credit. The authors therefore propose an output-based or performance-based resource allocation to compensate DHs for the additional costs due to increased surgical volume.

The recommendation for output-based resource allocation for surgery needs to be examined in the current Malawi health financing situation. Currently, Malawi spends approximately US$39 per capita per year on essential healthcare, a majority of which is from donor funding.^15^ For the DHs to increase their surgical volume and reduce referrals sustainably, the reimbursement for surgical services must be increased to match costs and overcome the financial disincentive to operate at the DH. While Broekhuizen et al consider the cost of scaling up the mentoring intervention in their model, they do not consider the additional costs of increased surgical volume and the cost of adequate reimbursement, which likely far outstrips the cost of scaling up the mentorship program alone.^1^ Particularly in a resource-constrained setting, calculation of these additional costs will be crucial to informing policy decisions.

 As well as the disincentive to reduce referrals for the DHs, the authors also acknowledge that for the CHs, the immediately noticeable effect of the program may be the absence of mentors. In contrast, the reduced bed days which may result from decreased referrals may be subtle and not be attained until a year or more into the program. The authors have not suggested how these disincentives to the mentorship program are to be overcome. It is commendable of the authors to be so open about this limitation. A clear strategy to incentivize increasing surgical capacity at the DH and incentivize the mentorship program at the CH is required if the program is to work sustainably.

## Governance

The SURG-Africa program consisted of mentorship of non-specialists to expand the surgical volume and decrease referrals to CHs in Malawi. The program, therefore, reinforces the important role of task-sharing/-shifting in increasing the surgical workforce density in LMICs and Malawi.^16,17^ However, as in many other contexts, this brings its own ethical challenges. Training of physicians is routed in basic sciences starting from undergraduate level up to specialisation. Non-physician training, including this mentorship program, lacks this component and concentrates on skills building. Most clinical decision making builds on an understanding of the basic sciences and pathology. This may be deficient in a skill-based mentorship program. However, once non-physicians have been trained to conduct some surgeries, there becomes a thin line on what they can do, and what their limits are. The Malawian legislator therefore needs to establish clear guidelines to make sure that non-physicians do not go beyond their scope of work but also that there is accountability. Should a non-physician clinician follow a surgeon’s recommendation if they disagree with it and are at risk of litigation even if they follow the surgeon’s recommendation? Given the unsolved questions around governance in task shifting, it is difficult for the Malawian patient to truly consent to their treatment without knowing who is ultimately responsible for their care. Therefore, the ethical and legal frameworks around task-shifting must be considered as interventions focused on scaling up task-shifting/sharing are considered in national policies to improve access to surgical care. In Malawi, task-shifting/sharing has been successfully used to scale up the treatment of other conditions such as HIV/AIDS.^18,19^ Lessons from task-shifting for such conditions, as well as lessons in surgical task shifting from other countries, could be considered in efforts to scale up the provision of surgical care by non-physician providers in Malawi.

## Quality of Care

 This study has chosen referrals from DHs to CHs and costs as the measurable endpoints to the SURG-Africa interventions. These endpoints have merits as they are easily quantifiable and measurable. However, neither of these reflects the quality of care. Access to surgical care is defined as access to care that is safe, timely, and affordable. These include clinical, process and implementation outcomes that should be taken into consideration in any surgical program. The authors determined a threshold of diminishing returns at which increasing the surgical volume in DHs will cost more than referrals to CHs. The concept of diminishing returns can also be applied to safety because running a DH at its maximum profitable capacity does not guarantee logistical efficiency, infrastructural capacity, or safety. Can we safely assume that other components of the Malawian health system would evolve to accommodate the increasing surgical capacity of DHs? Additionally, in any scenario which advocates for increased responsibility and capacity of task-shifted non-specialists, assurance of the quality of care must be central as increased surgical volume without quality assurance is not a real increase in capacity. Alongside mentorship, there must be a system of prospective monitoring of the quality of care outcomes for patients to ensure this intervention is safe and effective at reducing referrals.

## Conclusion

In summary, findings from the research conducted by Broekhuizen et al contribute to implementation research needed to sustainably scale up surgical interventions in resource-constrained settings.^1^ The policy options presented in their findings could inform the Ministry of Health policies to scale up mentoring of non-physician surgical providers in Malawi. In developing a policy to scale up mentoring, the additional cost of output-based resource allocation to DHs, as well as ensuring the quality of care and the complex governance issues around task shifting should be taken into consideration as these are paramount to the impact and sustainability of the proposed policy recommendations.

## Ethical issues

 Not applicable.

## Competing interests

 Authors declare that they have no competing interests.

## Authors’ contributions

 All authors contributed equally.
